# Teaching the ICF or using the ICF to teach? Lessons from first-time clinical practice

**DOI:** 10.4102/sajp.v82i2.2311

**Published:** 2026-04-30

**Authors:** Ilse du Plessis, Lunelle Pienaar, Heather Talberg

**Affiliations:** 1 Department of Health and Rehabilitation Sciences, Faculty of Health Sciences, University of Cape Town, Cape Town, South Africa; 2 Department of Health Science Education, Faculty of Health Sciences, University of Cape Town, Cape Town, South Africa

**Keywords:** International Classification of Functioning, Disability and Health, physiotherapy education, clinical reasoning, undergraduate students, early clinical placements, patient-centred care

## Abstract

**Background:**

Clinical education is central to physiotherapy training, yet many students enter their first placements with limited experience in holistic clinical reasoning. The International Classification of Functioning, Disability and Health (ICF) provides a biopsychosocial framework for understanding functioning, yet its use in early clinical education remains underexplored.

**Objectives:**

This study aimed to investigate second-year physiotherapy students’ self-reported understanding and application of the ICF following first time clinical exposure within a redesigned clinical course.

**Method:**

A quantitative cross-sectional descriptive study was conducted. All second-year physiotherapy students completing their first clinical placements in 2023 and 2024 at a South African university were invited to complete a self-developed survey. The survey assessed perceived understanding and application of ICF components using Likert-scale and categorical items. Descriptive statistics were used for analysis.

**Results:**

Of the 138 eligible students, 106 responded (76.9%). Students reported improved perceived understanding of the ICF, with retrospective ratings increasing from a mean of 6 before placement to 9 after placement. Confidence in applying the ICF was high (mean = 8).

**Conclusion:**

Early clinical exposure structured around the ICF may support the development of holistic reasoning and encourage students to move beyond impairment-focused thinking.

**Clinical implications:**

Embedding the ICF into early placements may strengthen patient-centred clinical reasoning.

## Introduction

Clinical education and workplace learning are foundational elements in health professions training, facilitating the transition from theoretical, classroom-based learning to real-world practice, and equipping students with the knowledge, skills and attitudes needed to address complex patient needs in a holistic, biopsychosocial manner (Patton, Higgs & Smith 2013; Spencer 2003). The International Classification of Functioning, Disability and Health (ICF), developed by the World Health Organization (2001), provides a comprehensive, internationally recognised framework for understanding health, functioning and disability at both individual and population levels. The ICF establishes a common systematic approach for evaluating body functions, activities, participation, environmental influences and personal factors, emphasising that functioning is shaped by multidimensional interactions (Barradell & Scholten 2025; Maart & Sykes 2022). In doing so, ICF provides a common language and structured framework for identifying impairments, activity limitations, participation restrictions and the influence of environmental and personal factors on health outcomes (Snyman, Von Pressentin & Clarke 2015; Sole et al. 2019).

The inclusion of the ICF framework in entry-level physiotherapy curricula is widely supported as a means of shifting from an impairment-centred model of care to one that is person centred and aligned with the biopsychosocial paradigm (Aftenberger & Taxer 2024; Barradell & Scholten 2025; Luft et al. 2022; Scholten, Ross & Bickford 2019). Its adoption in physiotherapy education has been associated with improved holistic assessment, interprofessional communication and context-sensitive intervention planning across diverse healthcare settings (Kloppers et al. 2015; Moran et al. 2020; Nguyen et al. 2016). In South Africa, the relevance of the ICF is further underscored by its formal recognition as the national standard for describing functioning, disability and health, following publication in Government Gazette No. 51362 in October 2024 (South Africa 2024).

Despite widespread endorsement of the ICF in health professions education, evidence on how the framework is integrated into undergraduate teaching and learning, particularly as a tool to guide early clinical reasoning, remains limited (Bornbaum et al. 2015). Emerging work suggests that structured ICF-informed curricula may influence graduates’ professional practice and clinical reasoning processes (Sandborgh et al. 2020), with particular relevance for gathering, interpreting and synthesising patient information to inform assessment and intervention planning (Snyman et al. 2015; Sole et al. 2019). The ICF has also been recommended as a valuable framework for uni-professional and interprofessional education, supporting shared understanding and collaborative practice across disciplines and stages of learning (Kloppers et al. 2015; Moran et al. 2020; Nguyen et al. 2016). However, less is known about how novice students operationalise the ICF during their first clinical encounters, particularly in relation to moving beyond impairment-focused reasoning towards activity- and participation-oriented perspectives.

At the University of Cape Town, the Clinical Physiotherapy I course provides second-year physiotherapy students with their first exposure to clinical practice. The course is structured to support the transition from classroom-based learning to supervised patient interaction.

The Clinical Physiotherapy I course structure was explicitly redesigned to align with both institutional and divisional curriculum renewal processes. This process of review and change formed part of the Educational Leadership Fellowship, a faculty development programme aimed at advancing curriculum innovation and leadership capacity within the Faculty of Health Sciences. This educational innovation involved changes to the clinical exposure component of the Physiotherapy I course – shifting it from short, discipline-specific rotational placements to sustained, longitudinal clinical experiences. The restructured learning approach aimed at facilitating a deeper integration of ICF concepts and encouraging students to move beyond impairment-based thinking towards holistic, patient-centred problem-solving and rehabilitation.

The first semester emphasises foundational theory with an emphasis on communication skills, ethical practice and the application of the ICF as a framework for understanding patient functioning, through interactive teaching sessions. The second semester comprises longitudinal, group-based clinical exposure at selected healthcare sites, including general hospitals, outpatient clinics and elderly care facilities. Weekly tasks are aligned to clinical placement objectives and emphasise components in the subjective and objective assessment of a client, which can be linked to levels of the ICF (see Table 1).

**TABLE 1:** Summary of the redesigned International Classification of Functioning, Disability and Health – aligned clinical exposure.

Component	Description
Duration of longitudinal exposure	6 weeks per clinical block
Weekly contact time	2 h per week on the clinical site
Group size	6–8 students per group.
Student-to-educator ratio	One clinical facilitator per group (1:6–8).
Educational approach	Structured, scaffolded integration of ICF domains across weeks, progressing from single-domain focus to integrated clinical reasoning.
Week 1 focus	Participation restrictions, personal and environmental factors, subjective assessment and facilitated group discussion.
Week 2 focus	Activity limitations, facilitated activity assessment, structured discussion of findings.
Week 3 focus	Body structure and function (impairments), supervised impairment assessment, structured feedback discussion.
Week 4 focus	Integrated assessment across all ICF domains, structured presentation of patient cases using the ICF framework.
Weeks 5–6 focus	Comprehensive assessment and participation in treatment planning targeting multiple ICF domains, guided problem-solving in simple cases and reflection on learning.
Feedback structure	Facilitated group discussion in each session. Students presented identified impairments, activity limitations, participation restrictions and contextual factors. Educators guided structured feedback linking findings to ICF domains and goal formulation.

ICF, International Classification of Functioning, Disability and Health.

Clinical educators purposefully guided students in applying ICF principles across diverse patient populations, with structured feedback and peer learning embedded in each session. This context provided a rigorous environment for evaluating how novice learners internalise and operationalise the ICF as a clinical reasoning framework in the early phase of their professional development. Findings may inform future curriculum development for fostering comprehensive, contextually relevant clinical competence.

The educational redesign was conceptually guided by the ICF as a biopsychosocial framework and interpreted through experiential learning theory, which emphasises learning through authentic experience, followed by sense-making and conceptual integration (Kolb 1984). Although not all four stages of Kolb’s learning cycle were explicitly addressed in our study, the supervised clinical exposure provided students with opportunities to engage in authentic subjective and objective patient assessments using the ICF framework. Through problem–list discussions, as well as verbal feedback from peers and clinical educators, students were supported in reflecting on their experiences and linking these reflections to their growing understanding of the ICF. These elements are recognised in the literature as supporting the development of professional reasoning and integration of experience into clinical understanding (Schön 1983).

Our study appraises the students’ perspectives of their learning after structured ICF task-focused integration during supervised patient exposure on a second-year first-time clinical placement. Our study aimed to describe the second-year physiotherapy students’ self-reported understanding and application of the ICF framework following structured, ICF-aligned clinical exposure during their first clinical placements.

## Research methods and design

### Study design

A quantitative, cross-sectional descriptive design was employed to gather comprehensive information about the students’ perceptions and experiences in a structured manner (Slater & Hasson 2025). Although data were collected once at the end of the clinical placements, students provided retrospective pre-post ratings of their perceived understanding of the ICF before and after placement. This approach was used to minimise response-shift bias and to capture students’ reflections on learning after clinical exposure, rather than to measure longitudinal change. All second-year students who entered the clinical course for the first time in 2023 and 2024 were invited to participate. This manuscript was prepared in accordance with the Strengthening the Reporting of Observational Studies in Epidemiology guidelines for reporting cross-sectional studies.

### Study setting

Our study was conducted within the Physiotherapy Division, Department of Health and Rehabilitation Sciences at the University of Cape Town. The participants were second-year physiotherapy students enrolled in the Clinical Physiotherapy I course. This setting provided a controlled environment to observe the impact of the curriculum changes on the students’ understanding of the ICF.

### Data collection

A self-developed descriptive survey was designed to assess the impact of revised course outcomes on student learnings on the ICF. The survey instrument was developed by the primary researcher, with review and input provided by the research team. The survey included both Likert sliding scale items, which rated understanding from 0 (no understanding) to 10 (very good understanding), as well as closed-ended categorical items exploring students’ perceived application of ICF domains during specific clinical assessment tasks. The instrument was developed for formative educational evaluation purposes and was not subjected to formal psychometric validation.

Participation was voluntary, data were anonymised at the point of collection and all responses were treated as confidential.

Second-year physiotherapy students were invited to participate in our study via a detailed information leaflet and informed consent form. Students who agreed to participate completed the self-administered survey in hard copy at the end of the second semester, after finishing their two clinical rotations. To minimise any potential undue influence, the authors were not present during the distribution or completion of consent forms and questionnaires; instead, the process was managed by a neutral colleague. All surveys were completed anonymously, and there was no penalty for non-participation.

### Data analysis

The collected data were captured in Excel and analysed by using Statistica version 14.0 (TIBCO Software Inc., 2020, California, United States [US]). Likert-type sliding scale responses (0–10) were treated as approximate interval data and summarised by using means and standard deviations to describe central tendency and dispersion. Each variable was analysed by using all available responses. Missing data were handled by using available-case analysis, with sample sizes varying across items. As our study was descriptive in nature, no inferential statistical testing was performed, and results are reported by using descriptive statistics only.

### Ethical considerations

Ethical approval was obtained from the University of Cape Town, Faculty of Health Sciences’ Human Research Ethics Committee (FHS HREC) (number 218/2023). Subsequently, permission to approach students was obtained from the university’s Department of Student Affairs.

## Results

Between 2023 and 2024, 138 students completed the second-year clinical course, with 106 (76.9%) responding to the survey across the two cohorts.

Students were each exposed to two clinical settings in the duration of the course, with general hospitals and elderly care facilities making up the bulk of the exposure (Table 2).

**TABLE 2:** Clinical exposure settings.

Placement site	2023 (*n* = 60)		2024 (*n* = 46)		Combined (*n* = 212 exposures)
	*n*	%	*n*	%	*n*
General hospital site	59	98	44	95.7	103
Elderly care facility	42	70	36	78.3	78
Community health centre	15	25	-	-	15
Paediatric site	-	-	4	8.7	4
Not specified†	4	6.7	8	17.4	12

Note: Percentages are calculated based on a total of two clinical placements, and ‘Combined (*n* = 212 exposures)’ refers to not-unique students (as students attended more than one placement site).

† , Not specified refers to surveys in which students did not indicate the specific placement site attended.

Students reported on their understanding and clinical application of the ICF pre- and post-block exposure, showing higher self-reported perceived understanding and application of the ICF following the clinical placement, with mean scores increasing from 6 before clinical blocks to 9 after placement.

Self-reported comfort in the clinical application of the ICF was high, with a mean score of 8.0 in both cohorts.

In addition, the students’ perceived understanding of the individual ICF components, including impairments, activity limitations, participation restrictions, environmental and personal factors, barriers, and facilitators, was consistently strong, with mean scores ranging from 9.0 to 10.0 (Table 3).

**TABLE 3:** Students’ self-reported retrospective pre-post ratings on their understandings of the International Classification of Functioning, Disability and Health.

Survey component	2023			2024		
	Results		Range	Results		Range
	Mean	s.d.		Mean	s.d.	
Comfort in the clinical application of the ICF	8	1.1	6–10	8	1.3	5–10
**Understanding of ICF components**						
Impairments	9	1.2	5–10	9	1.5	5–10
Activity limitations	9	1.2	5–10	9	1.8	5–10
Participation restrictions	9	1.1	5–10	9	1.1	7–10
Environmental factors	10	1.2	5–10	9	0.9	7–10
Personal factors	10	1.0	6–10	9	1.2	5–10
Barriers	10	1.0	6–10	9	1.1	5–10
Facilitators	10	1.0	6–10	9	1.1	6–10

ICF, International Classification of Functioning, Disability and Health; s.d., standard deviation.

Students were asked to indicate which specific levels of the ICF were particularly enhanced through a range of weekly tasks given for subjective and objective clinical assessment (Table 4). Students could select multiple ICF levels per task (so percentages do not sum to 100%).

**TABLE 4:** Enhancement of the understanding of international classification of functioning, disability and health levels related to aspects of physiotherapy assessment.

Assessment tasks	2023							2024						
	Total responses (*n*)	Impairment		Activity		Participation		Total response (*n*)	Impairment		Activity		Participation	
		*n*	%	*n*	%	*n*	%		*n*	%	*n*	%	*n*	%
**Subjective assessment**														
Client interview	60	42	70.0	53	88.3	55	91.7	46	24	52.2	38	82.6	41	89.1
**Objective assessment**														
Respiratory system (auscultation, palpation)	52	50	96.2	11	21.2	5	9.6	42	42	100.0	4	9.5	3	7.1
Musculoskeletal system (range of motion, muscle testing)	55	55	100.0	19	34.5	10	18.2	44	42	95.5	16	36.4	4	9.1
Gait assessment	56	43	76.8	43	76.8	26	46.4	43	34	79.1	27	62.8	15	34.9
Self-care	53	9	17.0	51	96.2	9	17.0	42	8	19.0	40	95.2	9	21.4
Transfers	53	20	37.7	43	81.1	12	22.6	45	15	33.3	39	86.7	11	24.4
Balance	54	38	70.4	46	85.2	23	42.6	42	31	73.8	30	71.4	9	21.4
Domestic activities	54	7	13.0	47	87.0	31	57.4	44	9	20.5	30	68.2	25	56.8
Functional mobility activities	60	25	41.7	51	85.0	34	56.7	44	15	34.1	37	84.1	21	47.7

Students could select more than one ICF level per assessment task; therefore, percentages may exceed 100%.

**FIGURE 1:**
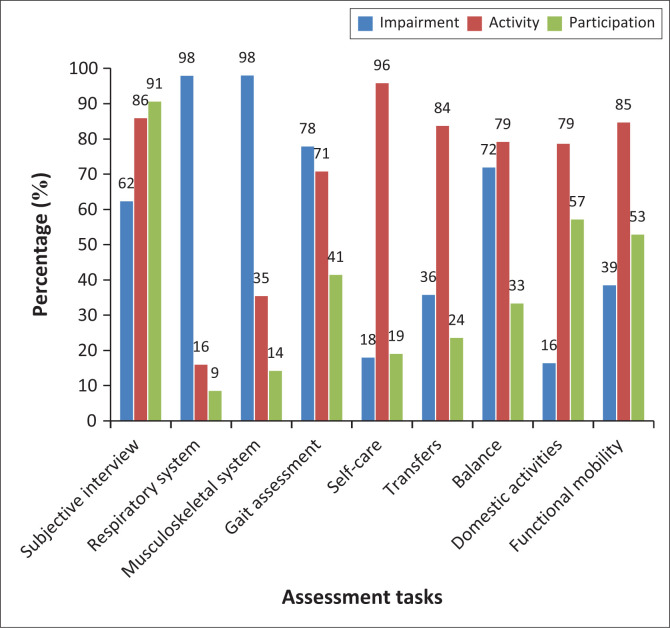
Alignment of assessment tasks to the International Classification of Functioning, Disability and Health framework.

Students indicated how different aspects of the subjective (patient interview) and objective assessment informed their understanding of the various levels of the ICF (Figure 1). The combined results from both cohorts (2023 and 2024) indicated that the subjective assessment enhanced their understanding across all levels of the ICF, with 91% indicating its value in establishing participation levels, 86% linking it to establishing activity levels and 62% gaining information on impairments through the process.

Assessment tasks strongly associated with impairments included the assessment of the respiratory (98%) and musculoskeletal system (98%), while activity limitations were most frequently identified in self-care (96%), transfers (84%), balance (79%), domestic activities (79%) and functional mobility activities (85%). Although these functional assessments were primarily linked to activity limitations, a substantial proportion of students still identified them as relating to impairments; for example, 39% of students associated functional mobility activities and transfers (36%) with the impairment level.

Participation was most linked to the assessment of domestic activities (57%) and functional mobility activities (53%) but was less frequently associated with other assessment tasks, such as gait (41%), transfers (24%), and self-care (19%).

**FIGURE 2:**
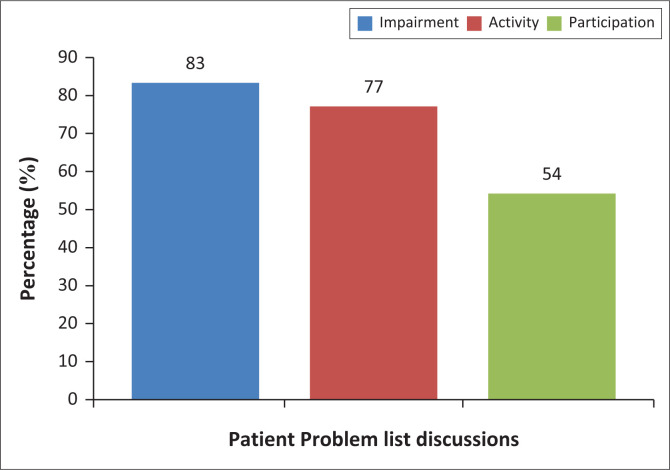
Role of problem list discussion in understanding the International Classification of Functioning, Disability and Health.

**FIGURE 3:**
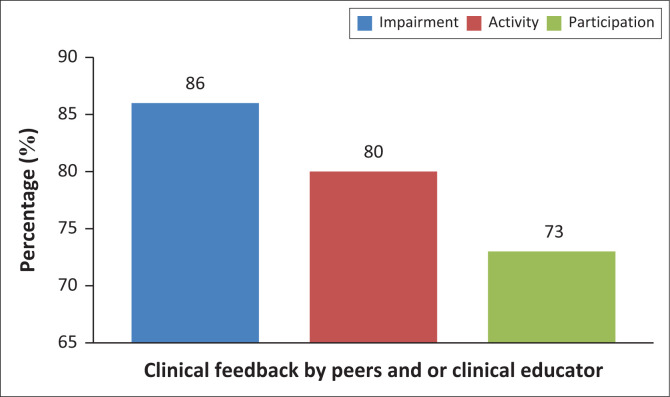
Role of clinical feedback in understanding the International Classification of Functioning, Disability and Health.

Both patient problem-list discussions (Figure 2) and clinical feedback (Figure 3) from peers or clinical educators, which happened as part of the clinical exposure, were identified by students as enhancing their understanding of all three levels of the ICF: impairment (83% – 86%) and activity (77% – 80%), while around half or more also associated these assessment components with participation (54% for problem-list discussions and 73% for clinical feedback).

## Discussion

Our study explored the integration of the ICF as a teaching framework for second-year physiotherapy students during their first clinical placements. Purposefully aligning learning outcomes and clinical tasks to the ICF aimed to scaffold the development of biopsychosocial clinical reasoning from the outset of students’ training, with the view that this step would, in the long term, better support the development of holistic clinical decision-making in students (Paltamaa et al. 2024; Scholten et al. 2021).

Retrospectively, students reported a high perceived understanding of the ICF prior to placement, with clinical exposure associated with higher retrospective ratings of understanding and confidence in applying the framework. This finding acknowledges the foundational theoretical integration of the ICF within physiotherapy courses as a teaching tool and conceptual framework to guide problem solving and thinking around holistic patient management at both the first and the second years of the programmes and suggests that follow-up authentic patient encounters are essential for bridging theory and practice (Bornbaum et al. 2015; Snyman et al. 2015).

The finding that subjective patient interviews and problem-list discussions were most strongly associated with perceived understanding across ICF domains highlights the importance of patient-centred information gathering needed for early clinical reasoning. This observation potentially implies an ability to obtain contextually relevant and function-related information from a patient even at this early clinical level. These interactive, patient-centred tasks appeared to help students contextualise impairments within broader functional and social participation domains, reflecting the value of communication-based approaches in fostering holistic thinking (Atkinson & Nixon-Cave 2011; Glassel & Hippold 2024; Miles et al. 2021). Such findings align with global evidence that shared frameworks like the ICF can accelerate the adoption of biopsychosocial reasoning and interprofessional communication skills among novice learners (Kloppers et al. 2015; Moran et al. 2020; Nguyen et al. 2016; Snyman et al. 2015).

By contrast, cardiorespiratory and musculoskeletal system assessments were most strongly associated with recognition of impairments, as nearly all students identified these components correctly in those assessments. While functional activities such as transfers, balance, domestic activities and mobility were reported to enhance understanding of both activity and participation, a notable proportion of students still primarily associated these tasks with impairments, instead of linking them consistently to activity and participation domains. This result suggests that while students can observe functional task performance, they may still interpret the task outcomes through a reductionist, biomedical lens, searching for underlying physiological deficits rather than considering the broader functional and contextual implications. This pattern is consistent with developmental models of clinical reasoning, in which early-stage learners anchor their clinical thinking in identifying discrete, measurable deficits (e.g. weakness, limited range, abnormal vital signs) and struggle to conceptualise functional performance as an emergent outcome shaped by multiple interacting factors (Abrandt Dahlgren et al. 2022; Cormack et al. 2023; Dalboni et al. 2023; Sole et al. 2019; Wijbenga, Bovend’Eerdt & Driessen 2019).

Several factors may account for this pattern. Firstly, curricular sequencing and assessment practices often emphasise the mastery of body structure and function examinations before functional and participation-level reasoning are introduced. As a result, students are more confident applying impairment-level frameworks to interpret observed movement than analysing the task in relation to activity or participation restrictions (Barradell & Scholten 2025; Gervais-Hupe et al. 2023). Secondly, the way clinical educators frame functional assessments and feedback may unintentionally reinforce this impairment focus. For example, when students observe difficulty with functional tasks such as transfers or mobility, feedback discussions often focus on underlying impairments, such as strength, balance or range of motion, as these are tangible and align with short-term treatment planning. While this approach supports skill acquisition, it may inadvertently obscure links among task performance, activity limitations and participation restrictions, particularly for novice learners (Gervais-Hupe et al. 2023; Wijbenga et al. 2019).

Additionally, novice students often lack the clinical experience to appreciate contextual influences, such as environmental barriers, social roles or personal factors, which may mediate task performance. Without this lens, students may see functional limitations as merely the sum of underlying impairments rather than as manifestations of activity or participation restrictions (Barradell & Scholten 2025; Scholten et al. 2019). This possibility is consistent with developmental models of clinical reasoning, which describe how students initially use rule-based, linear problem-solving and only gradually progress towards pattern recognition and holistic, context-integrated reasoning through guided experience and reflection (Abrandt Dahlgren et al. 2022; Sole et al. 2019). Until students gain repeated exposure to patients’ lived experiences and rehabilitation trajectories, they may find it difficult to mentally ‘scale up’ from component impairments to the broader activity and participation domains of the ICF.

This misalignment is educationally significant, as it can constrain the goal setting of students to impairment-focused outcomes and limit their capacity to design functionally meaningful, patient-centred interventions. Purposeful educational strategies are therefore required to explicitly bridge this gap, such as encouraging students to map each observed task to all ICF levels, embedding reflective discussions on contextual factors and progressively increasing the complexity of cases they are exposed to (Paltamaa et al. 2024; Reed et al. 2008; Scholten et al. 2019, 2021).

Furthermore, while students recognised participation issues during interviews, they appeared to lose this holistic framing when confronted with objective data. This loss may indicate a disconnect between information gathered subjectively and its integration into objective clinical reasoning, suggesting an area for further educational attention. Although students were introduced to the ICF conceptually, explicit ICF coding was not assessed or applied during clinical sessions, which may have contributed to difficulty in consistently distinguishing among impairment, activity and participation domains. Incorporating structured ICF coding exercises, alongside guided feedback and explicit mapping of assessment findings to all ICF levels, may therefore strengthen students’ ability to synthesise information and formulate goals that extend beyond impairments (Barradell & Scholten 2025; Paltamaa et al. 2024). However, as the stage of learning is only the second year, ideally, it may be better suited for a more advanced year of study, allowing a scaffolding of knowledge about ICF.

Overall, these findings reinforce the need for educator capacity-building on ICF-based teaching and supervision. Clinical educators play a pivotal role in modelling and reinforcing biopsychosocial reasoning and must be supported to facilitate students’ progression from the impairment-focused reasoning of a novice to a contextually grounded, patient-centred decision-making (Hess et al. 2025; Koh et al. 2023).

### Study limitations

Although students rated their understanding of the ICF before and after clinical placement, the cross-sectional design meant that individual pre-post changes could not be tracked; a longitudinal design would have provided stronger evidence of learning progression. As the survey was self-developed for formative course evaluation and not subjected to formal psychometric testing, findings should be interpreted as descriptive indicators of perceived understanding rather than a precise measure of ICF competence. The instrument primarily reflects alignment with course learning outcomes rather than an established measurement construct. The addition of open-ended qualitative questions could have offered deeper insight into how students conceptualised and applied the ICF in clinical reasoning. Furthermore, the perspectives of clinical educators, who play a central role in shaping students’ use of the ICF, were not included and could have provided valuable triangulation of findings. Finally, our study was limited to a single institution and year group, which may limit transferability to other educational contexts.

## Conclusion

As a preliminary study, these findings provide insight into how the ICF can be used to frame students’ initial exposure to clinical practice and problem-solving within physiotherapy education. Shifting clinical learning away from siloed, discipline-specific domains may support the development of more holistic perspectives on patient functioning, rather than an exclusive focus on disease or impairment. However, as novice learners, second-year students appear to require structured guidance to consistently move beyond impairment-based, task-oriented reasoning towards contextually relevant functional outcomes aligned with all domains of the ICF. Purposeful scaffolding and clinical educator support are therefore essential to support the development of higher-order clinical reasoning skills that integrate all ICF domains in patient-centred care.

### Novelty and contribution

This formative course assessment contributes to the growing understanding of how integrating the ICF into physiotherapy education can support the development of future healthcare professionals who deliver comprehensive, holistic care beyond the impairment level. The findings offer insights to guide curriculum development towards a broader, more consistent use of the ICF within clinical training.
